# Patient functional recovery after a 23-h surgery — a prospective, follow-up study

**DOI:** 10.1007/s00423-022-02502-y

**Published:** 2022-04-06

**Authors:** Ulla-Maija Ruohoaho, Sirpa Aaltomaa, Hannu Kokki, Maarit Anttila, Merja Kokki

**Affiliations:** 1 Department of Anaesthesiology and Intensive Care, Kuopio University Hospital, PO Box 100 FI-70029 KYS, Kuopio, Finland; 2 Department of Surgery, Kuopio University Hospital, Kuopio, Finland; 3 Institute of Clinical Medicine, University of Eastern Finland, Kuopio, Finland; 4 Department of Gynaecology, Kuopio University Hospital, Kuopio, Finland

**Keywords:** 23-H surgery, Postoperative recovery, Functional recovery, Short-stay surgery, Postdischarge symptoms

## Abstract

**Purpose:**

We evaluated patients’ functional outcomes 2 weeks after a 23-h surgery model in a tertiary care hospital.

**Methods:**

This prospective study comprised data on 993 consecutive adult patients who underwent a 23-h surgery. Patients were interviewed before surgery and at 14 days after surgery by telephone with a multidimensional structural survey including closed- and open-ended questions. Regarding functional outcomes, the patients were asked to assess their general wellbeing, energy levels and activities of daily living on a 5-point numeric rating scale (1 = poor to 5 = excellent). Data on patient characteristics, medical history, alcohol use, smoking status and pre-, peri- and postoperative pain and satisfaction with the care received were collected and analysed to determine whether these factors contributed to their recovery. The primary outcome measure was patient functional recovery at 14 days after surgery.

**Results:**

Most patients reported moderate to excellent functional outcomes: 93.6% (95% CI, 92.1–-95.1) of the patients showed a score ≥ 3 on the 5-point numeric scale. One out of four patients (23%) scored all three domains as excellent. A weak inverse correlation was noted between functional recovery and most pain in the 23-h postanaesthesia care unit as well as pain at 2 weeks after surgery. A weak positive correlation was noted between functional recovery and patient satisfaction with the instructions at discharge.

**Conclusions:**

Most patients showed ample functional recovery at 14 days after the 23-h surgery. Higher pain scores in the postanaesthesia care unit and 2 weeks after surgery predicted poor functional outcomes, and satisfaction with postoperative counselling predicted better outcomes.

**Trial registration:**

ClinicalTrials.gov NCT04142203.

## Introduction

An increasing number of surgical procedures are performed on a short-stay basis. In Finland in 1997, one-third (*n* = 88,000) of elective surgeries were performed on a same-day basis, in which patients are admitted, operated on and discharged during one working day without an overnight stay. In 2007, the proportion increased to 54% (*n* = 180,000). Since then, there has not been progress. In 2017, in Finland, 196,000 same-day surgeries were performed for 177,000 patients at a rate of 36 procedures/1000 inhabitants, representing 52% of total elective surgeries for that year [1]. The two driving forces increasing short-stay surgery are, first, the burden of increasing health care costs and, second, patient preference. Implementation of less invasive surgical interventions and better anaesthetics has improved patient safety and health outcomes. Shorter hospital stays after surgery have led to fewer complications and better outcomes [2, 3].

Not all surgical procedures and surgical patients meet the criteria for same-day surgery, and overnight admission may be preferred. A single overnight admission in a 23-h surgery model is a feasible method in cases where the surgical procedure or the medical, functional or social condition of the patient does not allow discharge on the day of surgery but prolonged ward admission is not needed [4]. As the proportion of same-day surgeries was relatively low in Kuopio University Hospital (KUH), representing 39% of elective surgeries (1), a new non-ward 23-h surgical unit was opened in May 2015 to increase the proportion of short-stay surgeries in the hospital [5].

Personalized information and both detailed verbal and written information instructions at discharge are known to enhance communication, give patients a sense of security at discharge and improve their self-management of their recovery postdischarge [6, 7]. In KUH, patient information about planned surgery is provided at the time of the operation decision. Postoperative verbal counselling at discharge is supplemented with written leaflets.

The information is operation-specific and includes (1) a general section about surgery and anaesthesia; (2) information on recovery, mobilization and restrictions after the specific type of surgery; (3) instructions for taking medication and pain killers; (4) red flag signs of abnormal healing or complications and (5) contact information/telephone numbers [8].

An important quality indicator for the success of short-stay surgery (hospital stay 24–72 h) is patient functional recovery after surgery. To assess the success of the implementation of the new 23-h surgery model in a tertiary care hospital, we planned this prospective follow-up study with the aim of evaluating postoperative recovery during the first 2 weeks after a 23-h model surgery. The primary outcome measure was patient functional recovery at 2 weeks after a 23-h model surgery, and the secondary outcomes were the factors affecting this recovery. Our study hypothesis was that the majority of the patients may grade their functional recovery as good or excellent. These data will be used to further improve patients’ perioperative care and counselling.

## Methods

**Fig. 1:**
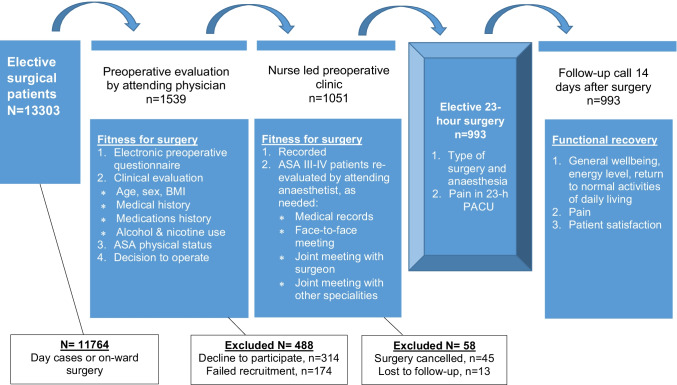
Study flow chart

This prospective follow-up study was approved by the Research Ethics Committee of the Northern Savo Hospital District, Kuopio, Finland (no. 73/2017, February 7, 2017) and had organizational approval. The 12-month study period was between May 16, 2017 and May 15, 2018, encompassing the second year of the new 23-h surgical unit. Consecutive adult patients aged 18 years or older (*n* = 1365) scheduled for the 23-h surgery in the hospital during the study period were asked to participate. We did not enrol patients with severe mental or neurodegenerative disorders, drugs or alcohol abuse or patients who were unable to understand Finnish or English. We failed to recruit 174 patients. The patients reached were given oral and written information on the study protocol, 314 declined to participate, and those who agreed to participate (*n* = 1051) provided written informed consent (see the study flow chart, Fig. 1).

This study is part of a large follow-up study of the implementation of a 23-h surgical model, patient satisfaction, quality of care and safety. Details of the implementation process, patient satisfaction and safety have been published elsewhere [4]. Here, we report patient functional recovery after the 23-h surgery.

In our hospital, the preoperative assessment is standardized. Patients are selected for the 23-h surgical model by an attending physician based on multidisciplinary agreed written criteria for this model. The surgeon makes the initial preoperative evaluation for patient fitness for surgery and records the American Society of Anesthesiologists (ASA) physical status classification [9] based on medical history, physical examination and data provided by the patient in an electronic preoperative questionnaire. At this visit/call, 2–4 weeks before their surgery, the patients are provided preoperative counselling. If a patient needs a more in-depth preoperative evaluation, the surgeon asks for an anaesthetist consultation in a preoperative clinic. For all patients, fitness for surgery was re-evaluated in a nurse-led preoperative clinic based on data in patient records. An attending anaesthetist reviewed all ASA III and IV patient assessments 1–2 weeks before the patient was scheduled to arrive for surgery. The patients with complex medical comorbidities have a face-to-face consultation with an anaesthetist, and higher‐risk surgical patients have shared decision‐making with anaesthetists, surgeons and other appropriate specialists. A 23-h unit nurse calls all 23-h surgery patients the day before surgery to confirm patient fitness for the surgery.

After surgery, the patients are admitted to a 23-h surgical model postanaesthesia care unit (PACU). In the hospital’s new PACU, there are separate sections with 12 beds designated for 23-h surgery patients. In our 23-h surgery model, the patients are discharged from the PACU by a 23-h unit nurse based on the approved discharge criteria after a one-night stay but before 10 am.

### Data collection

Patient characteristics and medical history data were collected from an electronic preoperative questionnaire, by phone during the preoperative call the day before surgery and by rechecking patient history on arrival at surgery and from patient electronic medical records (EMR, Uranus® and Oberon®, Consultants to Government and Industry, CGI®, Helsinki, Finland). We recorded patient age, sex, surgical speciality, ASA physical status and physical performance, body mass index (BMI), pain scores, anxiety and depression, concomitant diseases, medicine use and the use of nicotine and alcohol products. The patients’ nicotine use was classified into three categories: no use, regular/casual smoking/usage of other tobacco/nicotine formulations and ex-smoker. Alcohol consumption was graded according to the Current Care Guideline: no risk (women ≤ 7 doses/week, men ≤ 14 doses/week), moderate risk (women 7–11 doses/week, men 14–22 doses/week) and high risk (women 12–16 doses/week, men 23–24 doses/week). In Finland, one dose is defined as 12 g of alcohol [10].

Perioperative data were collected from the operative database records by Orbit® (CGI®) and Centricity Perioperative Anaesthesia™ (General Electric Healthcare, Helsinki, Finland).

Recovery data after discharge were collected by telephone interviews 14 days after surgery with a standardized questionnaire containing both closed- and open-ended questions. The assessment of patient functional recovery was based on asking about general wellbeing, energy levels and activities of daily living (ADL) functions e.g. standing, walking, eating and dressing, on a 5-point Likert scale (1 = poor, 5 = excellent). The patients were asked to evaluate their pain with an eleven-point numerical rating scale (NRS-11, 0 = no pain, 10 = most pain) at six time points: (1) before surgery, (2) first pain in the 23-h PACU, (3) most pain during the 23-h PACU stay, and pain (4) on walking, (5) on coughing and (6) at rest at 2 weeks after surgery. The interference of pain with ADLs was assessed with an NRS-11 (0 = does not interfere, 10 = completely interferes).

Postoperative complications and contacts to health care facilities after discharge were collected in the interview and by searching the patient EMRs.

Patient satisfaction with five phases of the 23-h process was evaluated with an NRS-11 (0 = totally dissatisfied, 10 = totally satisfied): (i) the preoperative visit in the surgical outpatient clinic and operative decision-making, (ii) preoperative planning and counselling by specialized perioperative nurses; (iii) operative treatment; (iv) postoperative care in the 23-h PACU and (v) counselling for postoperative care and rehabilitation at discharge. During the interview, patient information was computerized into an electrical database (Surveypal®, Tampere, Finland).

At discharge, the patients were asked whether they would recommend the service they used to their friends and family (The Friends and Family Test, FFT [11]. They were also encouraged to give open feedback. At discharge, the feedback query of the overall experience was performed using the Surveypal® platform with a tablet.

### Outcome measures

The primary outcome measure was patient functional recovery at 2 weeks after the 23-h surgery. For the secondary outcome, we evaluated potential factors that may predict delay or decrease in functional recovery.

### Statistics

Statistical analysis was performed using Statistical Package for Social Sciences Software (IBM SPSS Statistics 25, International Business Machines Corporation, Armonk, NY, USA). Categorical variables are expressed as the frequency and proportion (%) of patients. Continuous variables are expressed as the mean (SD) or the median, minimum and maximum as appropriate. Categorical variables were analysed by chi-squared tests, and continuous variables were analysed by the Mann–Whitney or Kruskal–Wallis test, as appropriate. For related samples, Friedman’s two-way analysis of variance was used. Pearson’s and Spearman’s correlation coefficients were used to measure the correlation between parameters. The *r*-values of − 0.19 to + 0.19 were considered to indicate very weak correlation; − 0.39 to − 0.2 or 0.2 to 0.39 was considered to indicate weak correlation; − 0.59 to − 0.4 or 0.4 to 0.59 was considered to indicate moderate correlation; − 0.79 to − 0.6 or 0.6 to 0.79 was considered to indicate strong correlation and − 1.0 to − 0.8 or 0.8 to 1.0 was considered to indicate very strong correlation. For the main outcome measures, 95% confidence intervals (CIs) were calculated. A *P* value < 0.05 was considered significant.

## Results

### Study population

**Fig. 2:**
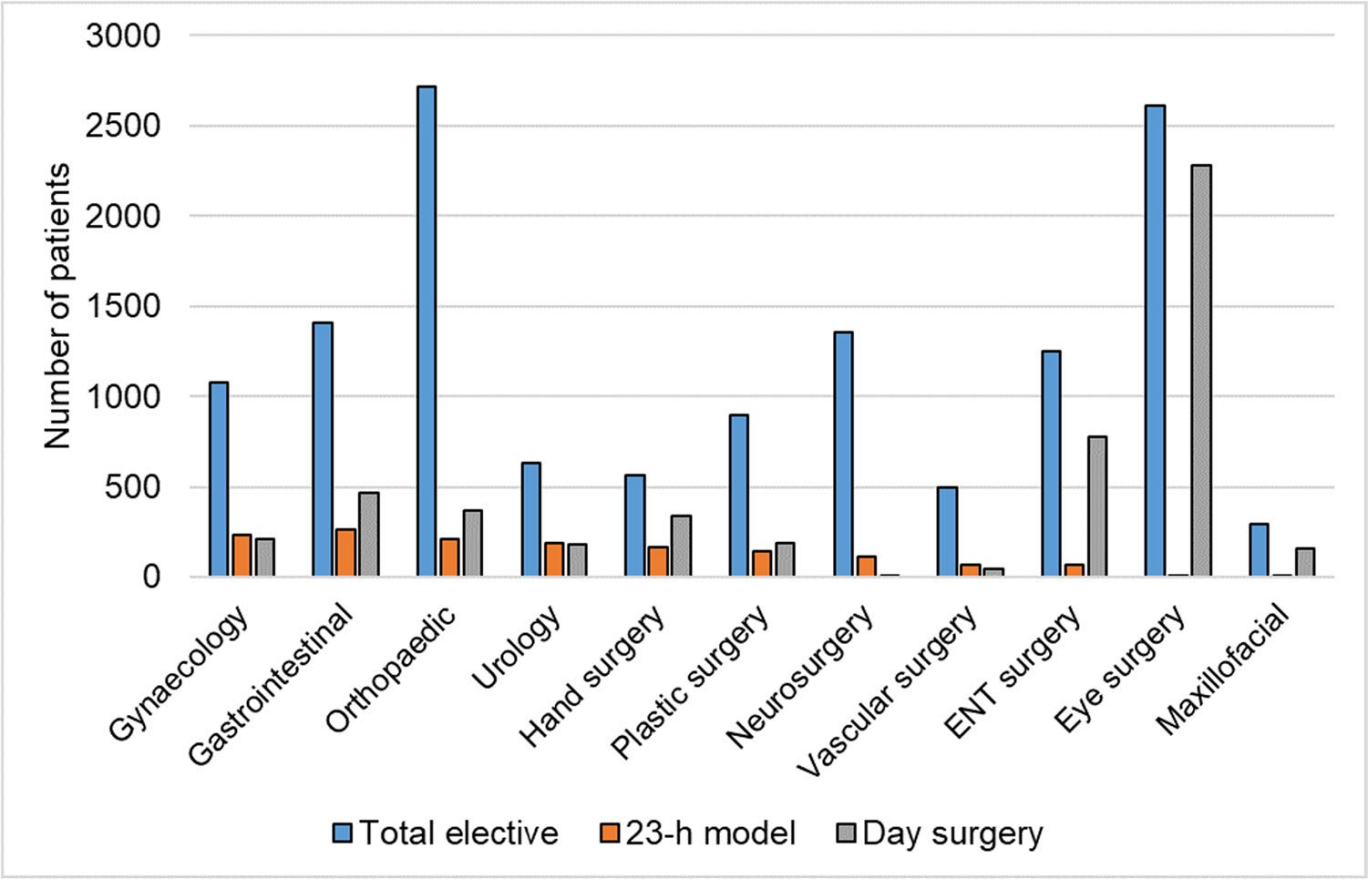
The total number of elective surgery patients and patients operated on in the 23-h model and day surgery in different surgical specialities in Kuopio University Hospital between May 16, 2017 and May 15, 2018. ENT, ear, nose and throat

During the 12-month study period, 13,303 patients underwent elective surgery in KUH; 1539 patients were scheduled and 1462 (11%) patients underwent 23-h surgery. There was a wide variation in the proportion of elective surgical patients selected for the 23-h model across the surgical specialities. Among hand surgery and urology patients, 29% of elective surgery patients underwent 23-h surgery, and among gynaecology patients, 22% of elective surgery patients underwent 23-h surgery. In contrast, only a few eye and maxillofacial surgery patients underwent 23-h surgery (Fig. 2). The proportion of day surgery and 23-h model surgery at the end of 2018 had increased to 48% of elective surgeries in the hospital (Fig. 2).

One thousand fifty-one 23-h surgery patients agreed to participate in the study. However, 13 subjects were lost to follow-up, and 45 cases were cancelled on the day of surgery. Thus, we included 993 patients (inclusion rate 71%) in the intention-to-treat analysis scheduled for elective 23-h surgery with eleven different surgery specialities (Fig. 1).

All the patients were interviewed at 2 weeks, for a response rate of 100%.

The patient characteristics are listed in Table 1. Most of the patients (60%) were women, and 70% (*n* = 669) were aged 18–64 years. One-third (35%) were overweight (BMI 25–29 kg/m^2^), and 29% were obese (BMI ≥ 30 kg/m^2^).

**Table 1:** Patient characteristics

Variable	Patients, *n* = 993
Sex, male/female, *n* (%)	398 (40%) / 595 (60%)
Age, years, mean (SD)	55 (15)
Physical status, ASA 1–2/3–4, *n* (%)*	812 (82%) / 181 (18%)
Body mass index, kg/m^2^, mean (SD)	27.6 (4.9)
Surgical specialty	
• Gynaecology, *n* (%)	175 (18%)
• Laparoscopic hysterectomy (*n* = 65), vaginal hysterectomy (*n* = 34); repair of cystocele or enterocele (*n* = 39); laparoscopic adnexal surgery (*n* = 32); other (*n* = 5)	
• Gastrointestinal surgery, *n* (%)	174 (18%)
• Cholecystectomy laparoscopic (*n* = 75), minilaparotomy (*n* = 5), hernia repair (*n* = 75), other (*n* = 19)	
• Orthopaedic surgery, *n* (%)	132 (13%)
• Shoulder arthroscopy incl rotator cuff repair (*n* = 65), lower limb arthroscopy (*n* = 36), lower leg or tarsal surgery (*n* = 21), other (*n* = 10)	
• Hand surgery, *n* (%)	120 (12%)
• Forearm and wrist incl arthroscopy(*n* = 98), upper arm and elbow incl arthroscopy (*n* = 22)	
• Urological surgery, *n* (%)	116 (12%)
• TURP and TUIP (*n* = 75), TURB (*n* = 24), other (*n* = 17)	
• Plastic surgery, *n* (%)	105 (11%)
• Reduction mammoplasty and reconstruction of breast (*n* = 56), mastectomy incl partial (*n* = 35), other (*n* = 14)	
• Neurosurgery, *n* (%)	80 (8%)
• Lumbar discectomy, decompression or laminectomy (*n* = 51), cervical discectomy (*n* = 27), other (*n* = 2)	
• Vascular surgery, *n* (%)	49 (5%)
• Partial thyroidectomy (*n* = 44), other (*n* = 5)	
• Ear, nose and throat, eye and maxillofacial surgery, *n* (%)	42 (4%)
• Ossicular chain surgery or cochlear implant (*n* = 18), tonsillectomy, laryngomicroscopy (*n* = 14), other (*n* = 10)	
Anaesthesia method	
• General anaesthesia, *n* (%)	564 (57%)
• Spinal anaesthesia, *n* (%)	249 (25%)
• Locoregional anaesthesia, *n* (%) Plexus (*n* = 141), regional anaesthesia (*n* = 39)	180 (18%)
Smoking or nicotine use, no/ex/current, *n* (%)	742 (76%) / 96 (10%)/144 (15%)
Alcohol, no/moderate/high risk use, *n* (%) **	657 (70%) / 242 (26%)/38 (4%)
Anxiety/depression, no/yes, *n* (%)	928 (93%) / 65 (7%)

^*^ASA American Society of Anaesthesiologists physical status classification (ASA, 2019); **alcohol consumption and risk use (7), *incl*. includes, *TURP* transurethral prostate electroresection, *TUIP* transurethral prostate incision, *TURB* transurethral bladder electroresection

### Functional recovery

The three functional recovery domains at 2 weeks after a 23-h model surgery are listed in Table 2. Functional recovery was moderate, good or excellent in most patients, and 929 of 993 patients (93.6%; 95% CI, 92.1–95.1%) graded all three domains of recovery, general wellbeing, energy levels and ADLs, with ≥ 3/5 on a 5-point Likert scale.

**Table 2:** The three components of functional recovery at two weeks after a 23-h model surgery. Data are number of cases (%). *N* = 993

Variable	Numeric rating scale, 0 = poor, 5 = excellent				
	Score 1	Score 2	Score 3	Score 4	Score 5
General wellbeing	2 (< 1%)	24 (2.4%)	237 (24%)	502 (51%)	228 (23%)
Having energy	2 (< 1%)	29 (2.9%)	238 (24%)	480 (48%)	244 (25%)
Activities of daily living functions	7 (< 1%)	57 (5.7%)	243 (24%)	393 (40%)	293 (30%)

### Factors that were correlated with functional recovery

The factors that were correlated with functional recovery are listed in Table 3.

**Table 3:** Factors that correlated with functional recovery. Data are Pearson’s correlation coefficient *r*-values

Variable	General wellbeing	Having energy	ADL functions
Pain			
• Most pain in 23-h postanaesthesia care unit	− 0.233	− 0.181	− 0.224
• At rest at 2 weeks	− 0.303	− 0.203	− 0.241
• During coughing at 2 weeks	− 0.307	− 0.260	− 0.211
• During walking at 2 weeks	− 0.372	− 0.306	− 0.274
Patient satisfaction with instructions at discharge	0.225	0.208	0.194

#### Pain

Half of the patients (566/990, 57%) had pain before surgery. Preoperative pain was mild in 224 (23%) patients and moderate or severe (NRS-11 score 4–10) in 332 (34%) patients with preoperative pain.

**Fig. 3:**
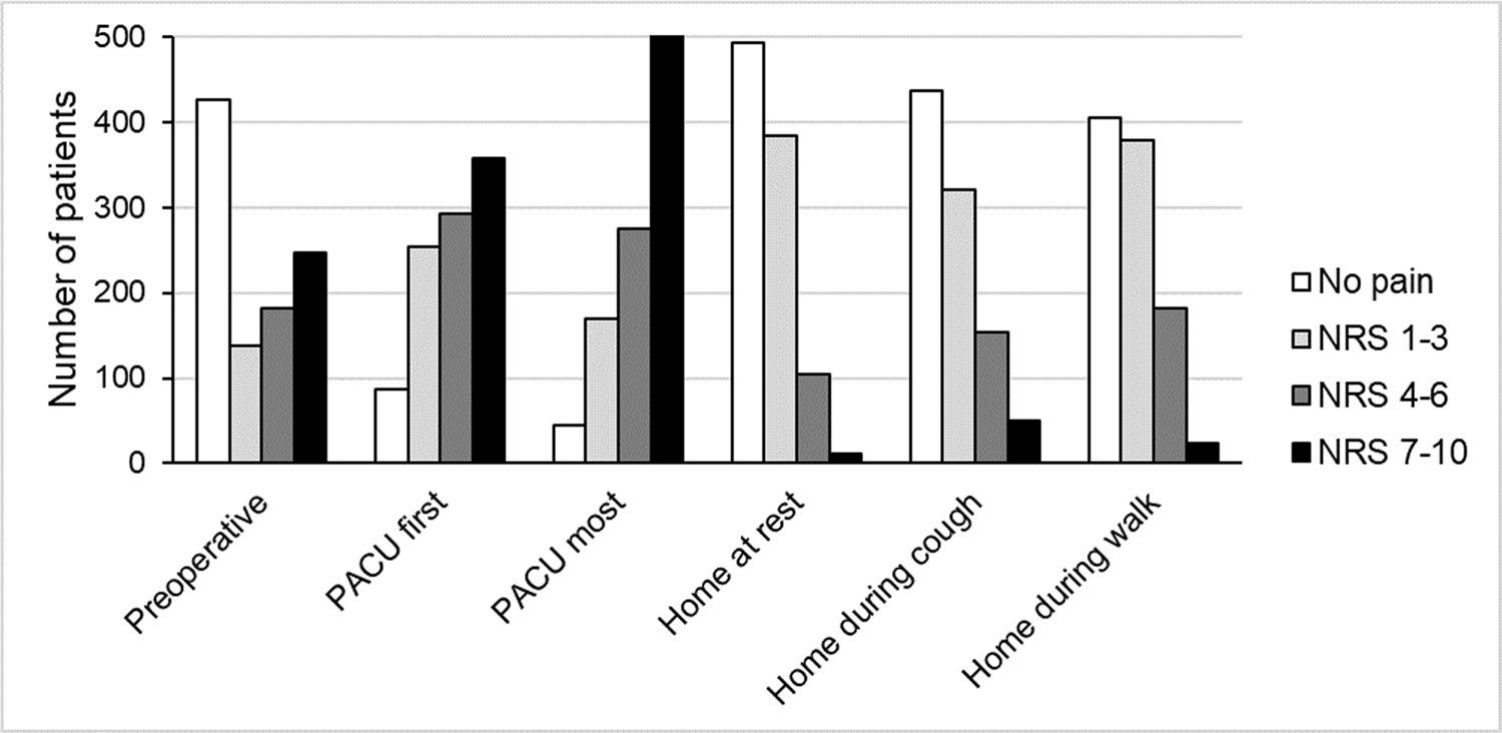
Patients reported pain scores with a numerical rating scale (NRS-11, 0 = no pain, 10 = most pain) at six time points: before surgery; first pain in the 23-h post anaesthesia care unit (PACU); most pain during the 23-h PACU stay; and pain at rest, during couching, and when walking at 2 weeks after the surgery

Pain was the most common postoperative adverse event. During the 23-h PACU stay, 948 (95%) patients experienced pain, which was moderate or severe in 779 (78%) patients (Fig. 3). Two weeks after surgery, 498 (50%) patients had pain at rest, and 115 (12%) patients had moderate or severe pain at rest. More dynamic pain was reported. During coughing, 525 (55%) patients experienced pain, which was moderate or severe in 207 (22%) patients. During walking, 583 (59%) patients experienced pain, which was moderate or severe in 205 (21%) patients.

A weak inverse correlation between the most pain during the 23-h PACU stay and general wellbeing and ADLs (*r* =  − 0.22 – − 0.23) was noted, and a very weak inverse correlation with energy levels (*r* =  − 0.18) was observed. A weak inverse correlation was also found between rest and dynamic pain at home at 2 weeks after the surgery and all three functional recovery domains, and the Pearson’s correlation coefficient values ranged between − 0.20 and − 0.37.

When asked about the interference of pain with ADLs, the mean score was low. The mean score on the NRS-11 was 2.2 (SD 2.3). However, in those with pain, the interference was substantial, as demonstrated by a moderate inverse correlation with general wellbeing (*r* =  − 0.41) and a weak correlation with having energy (*r* =  − 0.32) and ADLs (*r* =  − 0.33).

#### Patient satisfaction

**Fig. 4:**
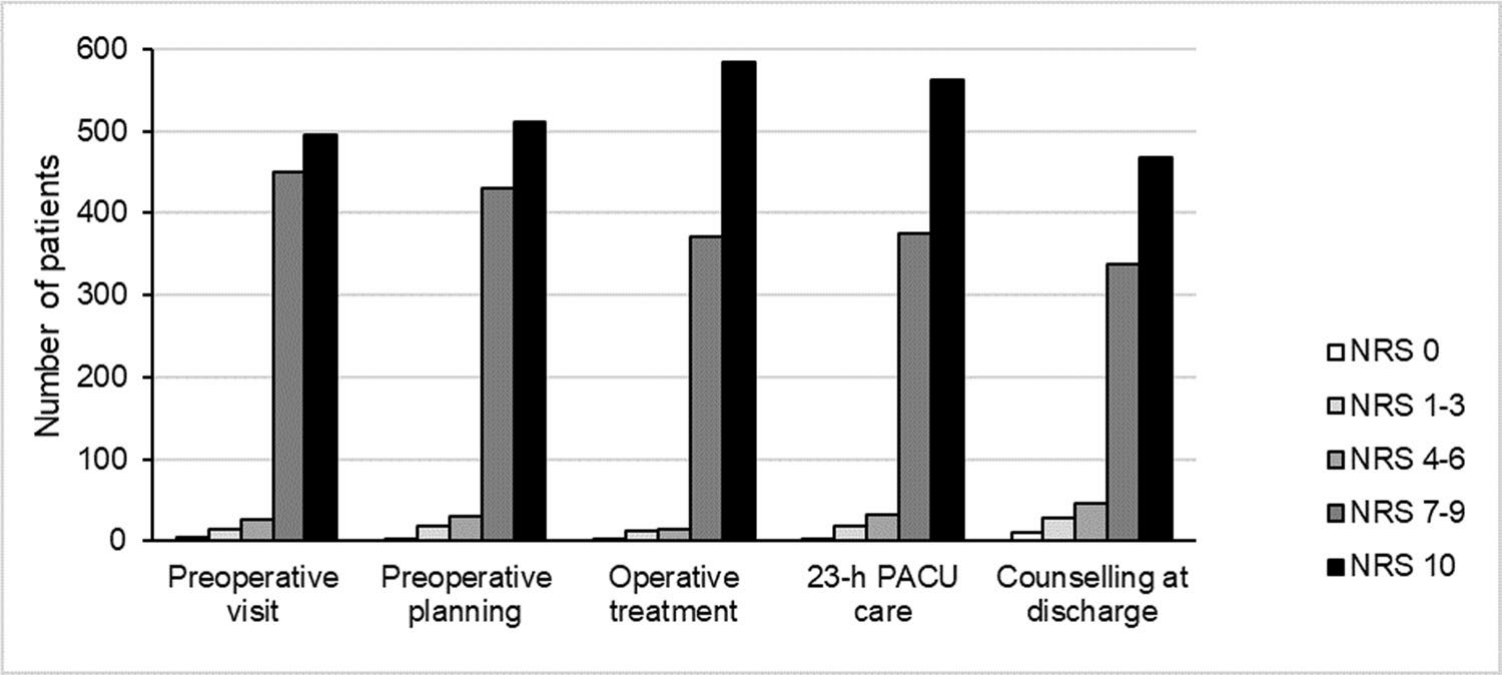
Patient satisfaction with the care on a numerical rating scale (NRS-11, 0 = totally dissatisfied, 10 = totally satisfied) at five time points: the preoperative outpatient clinic visit, preoperative planning and counselling, operative treatment, postoperative care in the 23-h post anaesthesia care unit (PACU), and counselling and instructions at discharge

Patient satisfaction with care was high when asked 2 weeks after surgery, but there was a significant difference in satisfaction between different states of care (*P* < 0.001) (Fig. 4). On the NRS-11, the mean patient satisfaction was 8.7 (SD, 1.6) for the preoperative visit in the surgical outpatient clinic, 8.7 (1.7) for preoperative planning and counselling, 9.0 (1.5) for operative treatment, 8.9 (1.6) for postoperative care in the 23-h PACU, and 8.5 (2.0) for postoperative counselling and instructions. Thirty-nine patients were not satisfied (NRS-11, ≤ 3) with the counselling and instructions at discharge compared to 16 patients with low satisfaction with the operative treatment and 19 patients with low satisfaction with preoperative visits.

Patient satisfaction with counselling at discharge had a weak positive correlation with general wellbeing and energy levels (*r* = 0.21–0.23) and a very weak correlation with ADLs (*r* = 0.19).

### Factors that were not correlated with functional recovery

Most variables had only a very weak correlation with functional recovery after the 23-h surgery at 2 weeks. The variables with the highest *r*-values included preoperative pain and ADL functions at 2 weeks after surgery (*r* =  − 0.18), postoperative complications (*r*-values − 0.199 to − 0.076), and patient satisfaction with preoperative counselling, operative treatment and postoperative care in the 23-h PACU, with *r*-values ranging between 0.12 and 0.19 (Table 4).

**Table 4:** Factors that did not correlate with functional recovery. Data are Pearson’s and Spearman’s correlation coefficient *r*-values as appropriate

Variable	General wellbeing	Having energy	ADL functions
Sex	− 0.003	− 0.053	0.025
Age	− 0.037	− 0.083	0.113
ASA physical status*	− 0.115	− 0.101	0.039
Body mass index	− 0.019	− 0.045	0.036
Preoperative pain	− 0.083	− 0.042	− 0.178
First pain in 23-h PACU	− 0.167	− 0.138	− 0.196
Mode of anaesthesia	0.032	0.041	0.104
Surgical speciality	0.091	0.083	0.090
Anxiety/depression	− 0.080	− 0.141	− 0.105
Smoking status	0.001	0.015	0.024
Alcohol consumption	0.007	0.074	0.021
Postoperative complications	− 0.199	− 0.173	− 0.076
After discharge contact to health care facilities	− 0.148	− 0.124	− 0.063
• Failure of 23-h process	− 0.077	− 0.067	− 0.003
Patient satisfaction with care			
• Preoperative visit	0.116	0.089	0.106
• Preoperative instructions	0.157	0.119	0.144
• Operative treatment	0.189	0.159	0.149
• Postoperative care in 23-h PACU**	0.192	0.186	0.138

^*^ASA American Society of Anaesthesiologists physical status classification (ASA, 2019); **PACU postanaesthesia care unit

In contrast, patient sex, age, ASA physical status, BMI, mode of anaesthesia, surgical specialty, anxiety/depression, smoking status and alcohol consumption only exhibited a negligible correlation with functional recovery (data not shown).

### The Friends and Family Test

The Friends and Family Test score for recommending the 23-h surgery services was high at 97.8%. In total, 545 out of 599 subjects would be extremely likely to recommend and 41 subjects were likely to recommend the 23-h surgery services to friends and family if they needed similar care. Four subjects were unlikely, and one was extremely unlikely to recommend our service; thus, the score for not recommending the 23-h surgery services was 0.8%.

## Discussion

This study assessed the patients’ perspective of recovery after surgery in a recently implemented 23-h surgery model in our hospital. After elective surgery and discharge the next morning, most of the patients returned to their ADLs at 2 weeks. Two weeks after surgery, three out of four patients reported their recovery as good or excellent in all three domains, including general wellbeing, energy levels and returning to the ADLs. The recovery was graded as poor or very poor in one or more of the three domains by 64 out of 993 patients (6%). Most pain experienced during the 23-h PACU stay, pain at 2 weeks after surgery, and patient satisfaction with the postoperative instructions and counselling were weakly inversely correlated with functional recovery. In contrast, no such correlation was found among surgical specialty, sex or age of the patients and postoperative functional recovery.

Consistent with gentle functional recovery, the patients’ feedback with the 23-h surgery care was very positive. The recommended FFT score was 97.8%, and the not-recommended FFT score was very low at 0.8%. This was similar to or higher than that of the hospital in general. During the same time period, the recommended FFT score for other services in our hospital was 96.0%, and the not-recommended FFT score was 1.5%. The willingness to promote our 23-h surgical model was also high in other published data. The National Health Service (NHS) in the UK is one of the most active to use FFT for driving service improvement. The NHS data in outpatient patients between May 2017 and April 2018 indicated that 93.6–94.1% would recommend and 2.4–3.3% would not recommend the NHS service [12]. The FFT measure is also utilized on a 0–100-point visual analogue scale from 0 = not at all likely to 100 = definitely recommend. For the NPS score (net promoter score), the cut-off value for promoters is 90 points or higher and that for detractors is 69 points or less. Stirling et al. evaluated FFT and NPS in 810 hand surgery patients at 14 months after surgery. On the NPS scale, 12.2% of hand surgery patients were classified as detractors, indicating that in long-term follow-up, the proportions of promoters would decline [13].

In the present study, to evaluate the quality of care, we used operative data as well as questionnaires in an electronic platform and interviews by telephone to incorporate patients’ voices into the development and measure the quality (patient-reported outcome measure) and experience (patient-reported experience measure) of care in the recently implemented 23-h surgery model in our hospital. During the last decade, instead of exclusively focusing on the length of stay, the patient’s perspective has been highlighted in the evaluation of health care performance. Commonly used care metrics include patient satisfaction and quality of life measures [14]. However, patient-centred care highlights a need for a shift in focus from patient satisfaction to patient experience and insights [15, 16]. Aspects such as functional status, pain, mobilization and basic activities of daily living are proposed outcome measures for evaluating the quality of surgery [16, 17].

Quality is also important from the perspective of health care providers, payers, administrators and policy-makers [16–18]. The evaluation of new investments or treatments compared with standard care should account for patient-reported functional health status and symptoms of the recovery period [16]. Traditional quality metrics, such as mortality, readmissions and complications, may not completely capture quality of care efficacy [16–18]. When changing the surgery process into enhanced protocols, such as 23-h surgery, it is important to measure the patient outcomes during implementation and in ongoing phases to improve services [15–17].

The organizational and operational perspective was the intention to shift the treatment paradigm from on-ward care and short-stay services towards day surgery with 23-h model input. The proportion of outpatient care and extended day surgery increased from 39% before the new 23-h unit was opened to 48% at 2 years after the implementation of the 23-h model (i.e. similar to that in other tertiary care hospitals in Finland) [1]. The current culture of shortening hospital stays is well tolerated and encourages the expansion of outpatient care and thus lower bed occupancy in operative wards.

Data on functional recovery after 23-h surgery are negligible. More data have been published on recovery after ambulatory and inpatient surgery [19–21]. However, data on ambulatory surgery cannot be directly employed for 23-h surgery, as the procedures are typically more extensive than those performed in ambulatory care. It is important to have data on different surgical pathways. In the present study, a higher ADL score was relevant when patients evaluated how they benefited from surgery. This was reasonable because if the surgical operation did not provide the expected benefit, the general well-being, energy levels and return to ADL could have been negatively affected.

Our data showing that postoperative pain and functional recovery contribute to patient satisfaction are consistent with recent data from Berkowitz et al. [15]. They published data on 10,000 surgical patients for the same time period as we had in the present study [15]. The patient characteristics and surgical procedures were quite similar in these two studies. However, in our study, all cases were elective surgery compared with 25% of emergency surgeries in the study by Berkowitz et al. In that study, moderate or severe postoperative pain also decreased patient satisfaction with surgical care and increased regrets after surgery. However, in contrast to Berkowitz’s study, postoperative complications exhibited only a very weak correlation with patient functional recovery in the present study. Moreover, less postoperative pain was reported in the present study, indicating that improvements in postoperative pain management may enhance quality of care and may improve the patient’s overall experience with the surgical process.

In the present study, the success of the 23-h model was high. This type of enhanced surgery pathway and early discharge could potentially increase patient contacts to primary health care/non-hospital health care system providers but to date have not been shown in fast-track surgery [22] or, as we reported earlier, in 23-h model surgery [4]. As we reported earlier, one-fourth of the patients contacted health care facilities after discharge, and 9% of patients visited either primary care or the hospital; the readmission rate (2%) and number of reoperations (6 cases) were low [4]. Fast postoperative recovery is essential to the success of the 23-h model. Delayed recovery causes unexpected harm, annoyance and costs to patients and organizations [2, 23]. Consistent with our data, prospective studies of early recovery after surgery (ERAS) pathways have demonstrated decreased length of stay with no change in readmission and even a decrease in complication rates and improved patient outcomes [2, 24].

To ensure health benefits and calm recovery, in patient counselling, it is important to encourage the patient to rehabilitate and return to normal daily living as soon as possible. Discharge counselling should focus on specific, patient-centred concerns and provide contact information in case of problems or uncertainty in recovery [8]. Hospitals may also use modern digital care pathways, specific cloud-based information technology platforms for digital service channels and open websites for special health care pathways, such as rehabilitation or surgery care [25], to facilitate patient advice on self-care and instructions and help with unexpected health problems. These services include online instructions and guidance available 24/7, chatbots, chats and symptom navigators that expand upon previous oral and written instructions [25].

There are limitations in the present study. The functional recovery assessment was based on patient self-reports. Although subjective recovery characteristics were assessed in a telephone interview, no objective measurement of functional activity was collected. The self-assessed return to ADL may not provide comprehensive information on recovery, as noted in a pilot study using wireless technology to assess recovery after abdominal cancer surgery [26]. Although patients in the Sun et al. study reported that they returned to the ADL, e.g. the number of daily steps they took was one-third of the preoperative number. In this case, the Hawthorne effect may also have an influence on patient-reported outcomes [27]. Patient-reported outcomes are highly subjective and easily influenced by predetermined expectations and motivation to please the researchers. Moreover, the follow-up time 2 weeks after surgery was short e.g. for evaluation of health-related quality of life. This type of data could provide more information about the benefits of 23-h surgery.

The strengths of the study were that multiple surgical specialties were studied, and consecutive patients in a normal clinical setting were enrolled. The inclusion rate of 71% is also considered appropriate to identify possible problems in the recovery trajectory. All the participants were also interviewed at 2 weeks after surgery, for a response rate of 100%. Thus, we believe our data are soundly based. The data can be used in the evaluation of pain treatments and patient counselling processes in the 23-h surgery model. Further steps in our institution are to strengthen the short-stay protocol and shift the flow to the 23-h model and day surgery. Further cost analysis of the 23-h surgery model will also be necessary.

In conclusion, postoperative functional recovery after different types of 23-h surgery was generally good. Severe postoperative pain in the PACU and pain at 2 weeks after surgery negatively affected functional recovery. Patient satisfaction with instructions at discharge presented a weak positive correlation with patient functional recovery.
